# Green Nanotechnology in the Formulation of a Novel Solid Dispersed Multilayered Core-Sheath Raloxifene-Loaded Nanofibrous Buccal Film; In Vitro and In Vivo Characterization

**DOI:** 10.3390/pharmaceutics13040474

**Published:** 2021-04-01

**Authors:** Sara Nageeb El-Helaly, Eman Abd-Elrasheed, Samar A. Salim, Rania H. Fahmy, Salwa Salah, Manal M. EL-Ashmoony

**Affiliations:** 1 Department of Pharmaceutics and Industrial Pharmacy, Faculty of Pharmacy, Cairo University, Cairo 11562, Egypt; sara.elhelaly@pharma.cu.edu.eg (S.N.E.-H.); rania.fahmy@pharma.cu.edu.eg (R.H.F.); salwa.salah@pharma.cu.edu.eg (S.S.); 2 Department of Pharmaceutics and Industrial Pharmacy, Faculty of Pharmacy, New Giza University, NewGiza, Km 22 Cairo-Alex Road, Giza 12588, Egypt; 3 Department of Pharmaceutics and Industrial Pharmacy, Faculty of Pharmacy, Al-Ahram Canadian University, 6th of October City 12556, Egypt; emansaadahmed136@gmail.com; 4 Nanotechnology Research Center (NTRC), The British University in Egypt (BUE), El-Sherouk City, Cairo 11837, Egypt; Samar.Salim@bue.edu.eg

**Keywords:** green nanotechnology, raloxifene, nanoemulsion, core-sheath nanofibers, response surface methodology, ex vivo drug permeation, in vivo pharmacokinetic studies

## Abstract

Green nanotechnology utilizes the principles of green chemistry to formulate eco-friendly nanocarrier systems to mitigate patients and environment hazards. Raloxifene (RLX) demonstrates poor aqueous solubility (BCS class II) and low bioavailability, only 2% (extensive first-pass metabolism). The aim of this study is to enhance RLX solubility and bioavailability via development of novel solid dispersed multilayered core-sheath RLX-loaded nanofibers (RLX-NFs) without the involvement of organic solvents. A modified emulsion electrospinning technique was developed. Electrospinning of an RLX-nanoemulsion (RLX-NE) with polymer solution (poly vinyl alcohol (PVA), hydroxypropyl methylcellulose (HPMC), and chitosan (CS) in different volume ratios (1:9, 2:8, and 4:6) using D-optimal response surface methodology was adopted. In vitro characterization of RLX-loaded NFs was performed; scanning electron microscope (SEM), thermal analysis, drug content, release studies, and bioadhesion potential. The optimum NFs formula was evaluated for morphology using high-resolution transmission electron microscopy (HRTEM), and ex vivo drug permeation. The superiority of E2 (comprising RLX-NE and PVA (2:8)) over other NF formulae was statistically observed with respect to Q60 (56.048%), Q240 (94.612%), fiber size (594.678 nm), mucoadhesion time 24 h, flux (5.51 µg/cm^2^/h), and enhancement ratio (2.12). RLX pharmacokinetics parameters were evaluated in rabbits following buccal application of NF formula E2, relative to RLX oral dispersion. E2 showed significantly higher Cmax (53.18 ± 4.56 ng/mL), and relative bioavailability (≈2.29-fold).

## 1. Introduction

The postmenopausal state is characterized by a sharp fall in the estrogen levels circulating in plasma. Such a fall is responsible for various symptoms: Hot flushes, mood swings, sleep disorders, as well as metabolic changes, including cardiovascular diseases and osteoporosis. These symptoms collectively affect postmenopausal women’s quality of life. Consequently, they add up to be the foremost cause of morbidity and mortality in postmenopausal women [1,2,3,4]. Raloxifene hydrochloride (RLX) is a selective estrogen receptor modulator (SERM) that has shown to preserve the beneficial effects of estrogen mainly increased bone mineral density (BMD) [5] and decreased LDL-cholesterol [6], and, at the same time, has an antiestrogen effect on mammary tissues and the uterus [7,8]. RLX prevents the transcriptional activation of genes containing the estrogen response element in reproductive tissue. In vitro studies showed RLX inhibits the estradiol-dependent proliferation of human mammary tumor cells [9]. Thus, RLX is used to prevent and treat osteoporosis and decrease the risk of developing invasive breast cancer in postmenopausal women. Although RLX is rapidly absorbed following oral administration (Approximately 60%), yet its absolute bioavailability is only 2% [10]. This is mostly because RLX undergoes extensive first-pass metabolism to the glucuronide conjugates raloxifen-49-glucuronide, RLX 6-glucuronide, and RLX-6, 49-diglucuronide [10]. As well, RLX belongs to class II drugs according to biopharmaceutical classification system (BCS), that is, low solubility and high permeability.

For bioavailability enhancement, many drug delivery systems have been adopted with the aim of efficient drug delivery to the plasma. As a rule of thumb, the right drug delivery system prominently participates in reducing side effects, improving patient’s compliance, and enhancing the bioavailability where controlling drug dissolution and absorption leads to improved therapeutic efficacy [11]. Nanotechnology, for example lipid microspheres, polymeric micelles, or liposomes, among many other manipulative techniques, tailor drugs at the nanosized range for nanomedicines development. However, there have been some worriment related to the potential harm these nanomaterials present [12]. The green synthesis performs simple, cost-effective, eco-friendly, with no organic solvents, and non-toxic final dosage forms [13]. Thus, our study aimed at formulating a buccal nanofiber (NF) film utilizing green nanotechnology as a tool to enhance RLX oral bioavailability with reduced potential hazards to the patient and the environment. 

Aiming to avoid first pass metabolism, various formulation techniques and routes of administrations have been adopted, however, buccal drug delivery is known for its high patient acceptability compared to other non-oral transmucosal routes of drug administration. The direct access to the systemic circulation through the internal jugular vein avoids acidic hydrolysis in the gastrointestinal tract (GIT) and bypasses the hepatic first pass metabolism enhancing the oral bioavailability. An added advantage is the rapid cellular recovery of the buccal mucosa after administration [14]. NFs offer the advantageous physical properties of the traditional polymeric films while maintaining the drug in the form of fibers in the nanometric size range, with a large surface area to volume ratio. Thus, it is being presented as a potent drug delivery vehicle with high permeability through mucosal membranes [15,16], flexibility in surface functionalities and superior mechanical performance (e.g., stiffness and tensile strength) [17]. Moreover, NFs has the benefit of preparing solid dispersions of a drug in a crystalline carrier which in turn increases its solubility and absorption [18]. NFs were prepared using the emulsion electrospinning method. It involves emulsification of active agents within the solution which is then dissolved in the appropriate solvents. Emulsion electrospinning has gained various levels of success in the production of drugs in the form of electrospun NFs for treatment of different diseases [19,20,21]. 

Different strategies have been made to improve RLX bioavailability: Murthy et al. [22] developed Self-assembled lecithin-chitosan RLX nanoparticles, Shah et al. [23] developed RLX nanostructured lipid carriers, Ağardan et al. [24] prepared RLX-loaded liposomes, Pandya et al. [25] prepared an RLX nanoemulsion, and Saini et al. [26] developed RLX-loaded chitosan nanoparticles.

Thus, the aim of this study was to formulate a novel solid dispersed buccal core-sheath RLX-loaded NFs through electrospinning of the oil in water raloxifene nanoemulsion (O/W) RLX-NE with polymer solution to improve RLX solubility, dissolution and, hence, bioavailability and use the benefits of buccal delivery to bypass the first-pass metabolism. An in vivo pharmacokinetic study of the buccal RLX-NF films compared to RLX oral dispersion was conducted in rabbits. 

## 2. Materials and Methods

### 2.1. Materials

Raloxifene HCl (RLX), USP 99.7% purity, batch number CFR018007 was purchased from BDR Pharmaceuticals International (Mumbai, India). Cremophor^®^ RH40 (polyoxyl 40 hydrogenated castor oil) was kindly provided by BASF SE (Ludwigshafen, Germany). Transcutol^®^ HP was a kind gift from Gattefosse (Lyon, France). Miglyol^®^ 812 (caprylic/capric triglyceride) was a kind gift from EIPICO for pharmaceutical industries (Cairo, Egypt). Hydroxypropyl methyl cellulose (HPMC), methocel™ K100LV (100 cP at a 2% addition rate in water), was purchased from Chempoint (Bellevue, WA, USA). Poly vinyl alcohol (PVA) (average Mw 146,000–186,000), chitosan (CS) high molecular weight (800–2000 cP), and acetonitrile were purchased from Sigma–Aldrich, Co. (St. Louis, MO, USA). Normal saline was purchased from Otsuka Pharmaceutical Co., S.A.E (Cairo, Egypt). Xylazine (XYLA-JECT^®^) was purchased from ADWIA Pharmaceuticals Co. (Cairo, Egypt). Ethyl acetate and formic acid were bought from El-Nasr Pharmaceutical Chemicals Co. (Cairo, Egypt). Olmesartan obtained from AstraZeneca (Cairo, Egypt). Potassium dihydrogen phosphate and disodium hydrogen phosphate were purchased from Sisco Research Laboratories (Mumbai, India). HPLC-grade methanol was purchased from Research-lab Fine Chem Industries (Mumbai, India). All other chemicals and solvents were of analytical grade and used as received.

### 2.2. Preparation of RLX-Loaded Nanofibers

#### 2.2.1. Preparation of RLX-Nanoemulsion

RLX-nanoemulsion was prepared using the Abd-Elrasheed et al. method [27]. Briefly, 5 mg RLX was vortexed first with 0.1 mL of Miglyol^®^ 812 (oil phase) for 1–2 min, then 0.4 mL of Cremophor^®^ RH40 (surfactant) and 0.4 mL of Transcutol^®^ HP (co-surfactant) were added and vortexed for 5 min, 0.1 mL of water was added dropwise while vortexing. Finally, homogenization was performed for 1 min at 10,000 rpm. RLX-NE was then sonicated for 10 min to eliminate any air bubbles.

#### 2.2.2. Preparation of Polymer Solutions

The choice of the polymers used was based on a preliminary study to choose the optimum type which could be electrospun in a continuous manner into NFs. PVA and HPMC were dissolved in double-distilled water to obtain 10% *w*/*v* and 1% *w*/*v*, respectively. For CS solution, CS was dissolved in 0.5% acetic acid to obtain a 1.5% *w*/*v* CS solution. All polymer solutions were stirred on a magnetic stirrer at 150 rpm and 80 °C for 1 h until complete dissolution, then stirring was continued at room temperature for 12 h. 

#### 2.2.3. Preparation of RLX-Loaded Nanofibers

Nine RLX-loaded NFs were prepared by the electrospinning technique comprising RLX-NE and different types of polymers with varied ratio. The composition of the developed RLX-NFs is shown in Table 1. The investigated variables were A: type of polymer (PVA, PVA + HPMC, PVA + CS), B: RLX-NE:polymer ratio (1:9, 2:8, or 4:6). For polymer solutions, fixed concentrations were used for each type and in case of polymer mixtures (i.e., PVA and HPMC, and PVA and CS) a 2:8 ratio were kept constant throughout the study as shown in Table 1. 

RLX-NE was stirred with polymer solutions at varied volume ratios as presented in Table 1 on a magnetic stirrer at 700 rpm for 30 min to obtain homogenous solutions followed by sonication for 90 min to get rid of any air bubbles before electrospinning. The homogenous solutions were then electrospun into NFs by electrospinner (NANON-01A, MECC, Fukuoka, Japan). The polymer solution was placed into 5 mL syringe (22 G). NFs were fabricated through electrospinning under optimized parameters set after preliminary experiments, and optimal spinning conditions were set to be: Applied voltage (25 KV), flow rate (0.9 mL/h), 15 cm distance between the nozzle and the aluminum foil collector and width (20 mm) [28]. All NFs samples were electrospun at ambient conditions with humidity of 48–50%. 

### 2.3. Characterization of RLX-Nanoemulsion 

#### 2.3.1. Determination of Particle Size (PS), Polydispersity Index (PDI), and Zeta Potential (ZP) 

The mean PS (Z-Average) and PDI of RLX-NE were determined at 25 ± 1 °C, in triplicates, via dynamic light scattering (DLS) analysis (Malvern Zetasizer Nano ZS; Worcestershire, UK). A 0.1 mL of the prepared RLX-NE formulation was diluted to 100 times with double distilled water. Following dilution, the glass tube was vortexed to ensure complete dispersion of the formulation. DLS analyzes the fluctuation of intensity of a scattered laser beam (caused by the Brownian random motion of droplets), at an angle of 90°, as a function of time [29]. The derived data were needed to estimate the diffusion coefficient and hence, the Z-average of droplets according to the Stoke–Einstein equation [30]. 

A laser doppler anemometer, coupled with the Zetasizer Nano ZS, was utilized to determine the electrophoretic mobility and hence, the zeta potential of the charged droplets.

#### 2.3.2. Drug Content

A total of 0.1 mL of the NE formulation was diluted with 140 mL of PBS solution, pH 6.8, and the absorbance of this solution was measured spectrophotometrically at 286 nm against a blank (0.1 mL plain NE mixed with 140 mL PBS solution pH 6.8) and the concentration was calculated.

#### 2.3.3. Spectroscopic Characterization of Percentage Transmission

A total of 0.1 mL of the NE formulation was diluted to 100 times with double-distilled water. The percentage transmission of the NE formulation after dilution was measured at 400–800 nm to determine the degree of clarity of the NE formulation.

### 2.4. Characterization of RLX-Loaded Nanofibers

#### 2.4.1. Solid State Characterization of RLX-Loaded Nanofibers

All RLX-NFs formulae were morphologically investigated using a scanning electron microscope (SEM) and subjected to thermal analysis, differential scanning calorimetry DSC, Fourier-transform Infrared Spectra (IR), and powder x-ray diffraction PXRD 

##### Scanning Electron Microscope (SEM)

The fiber surface morphologies of the RLX-NFs were assessed via a SEM (JEOL GSM6610LV, JEOL Ltd., Tokyo, Japan) operating at acceleration voltage of 30 kV. NFs samples were placed on the carbon tape. The fiber diameter was measured using Image J software from the SEM pictures in original magnification. At least 15 isolated NFs were randomly selected, and their diameters and diameter distributions were measured and averaged.

##### Differential Scanning Calorimetry (DSC)

The physical state of all RLX-NFs formulae and their individual components, as well as the pure drug, were performed using a Shimadzu differential scanning calorimeter (DSC-50, Shimadzu, Japan). Samples were placed in a standard aluminum pan and then heated at a constant rate of 5 °C/min under the atmosphere of nitrogen gas carrier in a temperature range from 20 to 350 °C.

##### Fourier-Transform Infrared Spectra (FT-IR)

FTIR of RLX-NFs was recorded by a Fourier transform infrared spectrometer (FTIR, Shimadzu FTIR-8400 S, Kyoto, Japan). The experiments were carried out in the range of 4000 to 500 cm^−1^. The infrared spectra were recorded in the transmission mode using thick mats of RLX-NFs.

##### Powder X-ray Diffraction (PXRD)

PXRD studies of RLX and all RLX-NFs were assessed at room temperature (PANalytical X’Pert PRO diffractometer; Almelo, Netherlands). The samples were prepared using nickel filtered Cu Kα radiation (λ = 1.542 Å, 45 kV, and 35 mA). The results were presented as intensity versus 2θ (5–80°) [31].

#### 2.4.2. Determination of Drug Content and Homogeneity of RLX-Loaded Nanofibers

Accurate weights from different sites of RLX-PVA-NFs, RLX-PVA-HPMC NFs were dispersed in double-distilled water and RLX-PVA-CS NFs was dissolved in 1% acetic acid by homogenization at 10,000 rpm. The drug content and the homogeneity of NF films were then analyzed using UV spectrophotometer. Measurements were performed, in triplicate, at room temperature. 

#### 2.4.3. In Vitro Release Studies

After drug content determination of all RLX-NFs formulae, samples of RLX-NFs equivalent to 1 mg RLX were accurately weighed and then, the RLX-NFs were immersed in 25 mL release medium (PBS, pH 6.8 of 0.1% tween) [32] in accordance with optimum sink conditions and gently stirred at 37 ± 1 °C with a speed of 50 rpm in a shaking water bath. At certain time intervals (10, 20, 30, 40, 50, 60, 120, 180, and 240 min), a certain volume of the solution (1 mL) was removed and replaced with the same volume of a fresh medium. Withdrawn samples were suitably diluted and analyzed using UV spectrophotometer and the mean drug released percentages (± S.D.) were plotted versus time [28].

#### 2.4.4. Bioadhesion Potential of RLX-Loaded Nanofibers

Adhesion study was performed to RLX-loaded NF films by adapting a method of Nakamura et al. [33]. 100 g of a hot agar/mucin solution (1 and 2% *w*/*w*, respectively, in phosphate buffer pH 6.8) were casted on a glass plate (10 cm × 10 cm) and left to gel at 4–8 °C for 3 h. The gel was then equilibrated for one hour to the test conditions of 25 °C and 75% relative humidity (saturated sodium chloride solution) in a chamber. NF films were placed on top of the gel, moved downward due to gravity after the glass plate was turned into a vertical position. The time taken to move from the top to the bottom of the plate was recorded (*n* = 3). 

### 2.5. Optimization of RLX-Loaded Nanofibers via D-Optimal Response Surface Methodology 

A response surface methodology was used for optimization using D-optimal design to study the effect of the different formulation variables on the characteristics of the developed RLX-loaded NFs using Design-Expert^®^ software (Stat-Ease, Inc., Minneapolis, MN, USA). The independent variables were, A: Type of polymer (PVA, PVA + HPMC, PVA + CS), B: RLX-NE:polymer ratio (1:9, 2:8, or 4:6), while the % drug released at time 60 min, Q60 (Y1), % drug released at time 240 min, Q240 (Y2), fiber size (Y3), and mucoadhesion time (Y4) were chosen as the dependent variables (responses). 

The desirability formula was adjusted to minimize fiber size and maximize Q60, Q240 and mucoadhesion time. One-way ANOVA and LSD post hoc analysis were attempted to compare the results of the selected responses using SPSS software 27.0 (SPSS Inc., Chicago, IL, USA). A *p*-value < 0.05 was considered statistically significant. 

Numerical optimization technique was chosen for optimization of the responses. This method is based on the utilization of desirability functions and the optimum RLX-loaded NFs was selected.

### 2.6. Characterization of the Optimum RLX-Loaded Nanofibers

#### 2.6.1. High-Resolution Transmission Electron Microscope (HRTEM) of the Selected RLX-Loaded NFs

The morphology of the selected RLX-NFs formula was evaluated by high-resolution transmission electron microscopy (HRTEM, JEM-2010, JEOL) with an accelerating voltage of 200 KV. TEM was operated at 15 kV, and the fiber samples were prepared by making a suspension of the NFs in ethyl alcohol and directly depositing the as-spun ultrafine fiber suspension onto copper grids.

#### 2.6.2. Ex Vivo Drug Permeation Studies

The protocol of the ex vivo permeation studies was approved by the institutional review board, Research Ethics Committee Faculty of Pharmacy, Cairo University (approval no. PI 2762). Cow buccal mucosa, without any treatment, was freshly obtained from a local slaughterhouse immediately after the animal was killed and stored on normal saline in an ice box until it was transferred to our laboratory [34]. According to the results of the in vitro characterization studies, freshly prepared RLX-suspension (2 mg/2 mL PBS pH 6.8 of 0.1% tween 80) and the optimum RLX-loaded NFs formula equivalent to 2 mg RLX, were exposed to permeation testing of the drug through cow buccal membrane using the method described by Tayel et al. [35]. The apparatus used to test the permeation consisted of a glass tube (1.3 cm diameter) opened from both ends. The membrane was stretched over one open end of the glass tube and made water-tight by a rubber band then loaded with the formula forming the donor chamber. Two milliliters of phosphate buffer, pH 6.8, of 0.1% tween was transferred to the donor chamber to simulate the conditions inside the buccal cavity. The tube was attached to the shaft of the USP dissolution apparatus. The tube was then immersed in phosphate buffer, pH 6.8, of 0.1% tween 80 contained in the USP dissolution apparatus flask so that the membrane was just below the surface of the recipient solution. The temperature was maintained at 37 ± 0.5 °C, and the apparatus was run at 100 rpm for 6 h. Samples of 0.5 mL were withdrawn at 0.5, 1, 2, 3, 4, and 6 h, and were compensated for by equal volume of fresh buffer. The withdrawn samples were analyzed using a validated LC/MS/MS method. The drug permeation studies were conducted in triplicate and the results were presented as mean ± SD. The cumulative drug amounts permeated through the buccal mucosa per unit area (μg/cm^2^) were plotted against time (h). The flux (Jss) at steady state was calculated. The enhancement ratio (ER) was calculated via dividing the flux value of the RLX-NFs formula by that of the aqueous dispersion, according to the following equations [36]:

J_ss_ = Cumulative amount of drug permeated/time × Diffusion area (1)

ER = Flux of RLX-NFs/Flux of aqueous RLX dispersion(2)

### 2.7. In Vivo Estimation of RLX Pharmacokinetic Parameters in Rabbits

#### 2.7.1. Study Design

The in vivo study was conducted, to compare the pharmacokinetics parameters of RLX-NFs following the buccal application of formula E2 (test treatment) and the oral administration of RLX aqueous dispersion (reference treatment). The study was performed using a non-blind, two-treatment, two-period, randomized, crossover design. The protocol of the in vivo study was approved (PI–2762) by the Research Ethics Committee at the Faculty of Pharmacy at Cairo University, Cairo, Egypt.

#### 2.7.2. Animals

Adult male New Zealand albino rabbits (weighing 3.0 ± 0.1 kg each) were derived from the animal house (Faculty of Pharmacy, Cairo University, Egypt). They were housed under optimum environmental conditions with respect to room temperature (25 °C), humidity (50%), ventilation (15–20 AC/h), and adoption of alternating 12 h light/dark cycles. The animals were accommodated as one rabbit per cage, fed with standard dry food, and had free access to water.

#### 2.7.3. Administration of Treatments and Blood Collection

A crossover design was conducted where the animals were randomly divided into two groups and each group contained three rabbits. The rabbits were anaesthetized using subcutaneous injection of xylazine HCL (20 mg/kg body weight) [37]. Then, a second dose of xylazine HCL was given after four hours to keep them sedated for 8 h to ensure proper application of the formulations and protect them till complete absorption of formulations. 

Group I administered the optimum RLX-NFs films (formula E2) through the buccal mucosa in a dose level equivalent to 2.64 mg/kg body weight [38], Group II administered RLX oral suspension [32] in the same dose level. Following a wash out period of eight days, the reverse of randomization of the treatments took place in phase II. Blood samples of (1–2 mL) were collected into the EDTA-added centrifuge tubes at the time points (0, 0.25, 0.5, 1, 1.5, 2, 4, 6, 8, 12, 24, and 48 h). Regular check was done on the NFs in the rabbits’ buccal cavity in order to check on their degradation pattern induced by the salivary flow. Blood samples were centrifuged at 4000 rpm for 15 min at room temperature. The plasma was separated and stored at −20 °C. Later RLX concentration in plasma was determined using a validated LC/MS/MS method. 

#### 2.7.4. Estimation of RLX Concentration in Rabbit Plasma

The concentration of RLX in the thawed plasma samples were analyzed using a triple quadrupole LC/MS/MS mass spectrometer (Micromass, Manchester, UK), according to the method developed by Said et al. [39]. Briefly, 200 μL of Olmesartan solution (internal standard; 100 ng/mL) was vortexed with 0.5 mL of plasma samples. The drug and the (internal standard) were extracted with ethyl acetate (4 mL), and vortexed for 1 min. The organic layer was separated by centrifugation (Eppendorf centrifuge 5804 R, Hamburg, Germany) at 4000 rpm for 10 min at 4 °C, and finally dried using a vacuum concentrator (Eppendorf 5301, Hamburg, Germany). The dried sample was reconstituted in 200 μL of the mobile phase, prior to analysis. For analysis, the samples (20 μL) were injected into the mass spectrometer. The elutes were separated on Agilent TMZORBAX Eclipse Plus (4.6 mm × 50 mm, 100 Å, 5 μm) using an isocratic mobile phase consisting of a mixture of 80% acetonitrile and 20% of 0.1% formic acid in water, at a flow rate 1 mL/min. The LC/MS/MS mass spectrometer was equipped with an electrospray ionization (ESI) source that was adjusted to operate in the positive ion mode. The multiple reaction monitoring mode was employed to detect the transitions of the m/z 244.0 and 315.96 precursor ions to the m/z 185.30 and 270.00 product ions for RLX and IS, respectively. A linear calibration curve (r2 = 0.9961) was constructed between RLX plasma concentrations (ng/mL) and RLX/IS peak area ratios over RLX concentration range of 1–300 ng/mL.

#### 2.7.5. Pharmacokinetics and Statistical Analyses

The mean RLX plasma concentrations (± S.D) of both treatments were plotted versus time. The pharmacokinetic parameters; peak RLX plasma concentration (C_max_, ng/mL), the time to reach C_max_ (T_max_, h), the mean residence time (MRT_0–∞_, h), the elimination half-life (t_1/2_), the area under the curve from zero to the last sampling point (AUC_0–48 h_, ng.h/mL) and from zero to infinity (AUC_0–∞_ ng∙h/mL) were estimated by application of non-compartmental analysis using WinNonlin software Ver. 8.3 (New Jersey, NJ, USA), and multivariate ANOVA using general linear model in SPSS software 27.0 (SPSS Inc., Chicago, IL, USA) and a non-parametric Wilcoxon signed rank test for Tmax. The AUC_(0–48 h)_ and AUC_(0–∞)_ values were used for calculating the relative bioavailability of the test treatment. Results were expressed as the mean (± S.D) except for T_max_ which was expressed as the median value. 

## 3. Results and Discussion

### 3.1. Preparation of RLX-Loaded Nanofibers

Herein, A novel buccal RLX-NFs were formulated via electrospinning of RLX-NE with polymer solutions that yielded a core-sheath NFs without the use of organic solvents, which paves the road to ‘‘green’’ drug delivery systems. Nine RLX-loaded NFs were successfully prepared via the electrospinning of RLX-NE with polymer solution (at NE:polymer volume ratios 1:9, 2:8, and 4:6) using (PVA, PVA + CS, or PVA + HPMC) polymer solutions as presented in Table 1. The incorporation of the drug in the form of O/W NE presents the drug into the NFs in a more soluble form. Hence, the drug diffusion is enhanced with subsequent efficient penetration, increased rate of drug absorption, and enhanced bioavailability [19,40,41]. 

### 3.2. Characterization of RLX-Loaded Nanoemulsion

#### 3.2.1. Determination of Particle Size (PS), Polydispersity Index (PDI), and Zeta Potential (ZP) 

It was observed that the prepared NE system has a globule size 62.60 ± 6.82 nm. The small mean droplet size (MDS) might be attributed to the use of the proper surfactant/Co-surfactant mixture which can be adsorbed around oil-water interface reducing the free energy of the system, giving a small globule size [42]. Such surfactant/co-surfactant mixture composition and ratio was designated based on thorough preliminary experiments.

Poly-dispersity index (PDI) values ranged from 0.122 to 0.127, these small values of PDI indicates a monodispersed population. RLX-NE showed a ZP value of (4.33 ± 0.31 mV). The significance of such low ZP was minimum because the RLX-NE system will be further incorporated into nanofiber formulations.

#### 3.2.2. Drug Content

The mean RLX content in the formulated NE system was found to be 100.34 ± 1.17% confirming the absence of drug loss during the formulating procedures.

#### 3.2.3. Spectroscopic Characterization of Percentage Transmission

Percentage transmission (%T) of diluted RLX-NE was measured spectrophotometrically in the visible range (400–800 nm) and found to be (99.86% T), indicating highly clear and stable system obtained by aqueous dilution of the prepared NE formula. This might be attributed to the fact that the oil droplets are in a state of tremendously fine dispersions as presented above by the miniature globule size [43].

### 3.3. Characterization of RLX-Loaded Nanofibers

D-optimal Response Surface methodology was employed to estimate the significance of the direct and/or interactive contribution of the formulation variables on the characteristics of the developed RLX-loaded NFs. One categorical factor (polymer type, A) and one numerical factor (NE: polymer ratio, B) were investigated. The results were statistically analyzed and the final equations for the four responses (Q60 min, Q240 min, fiber size, and mucoadhesion time) were derived in terms of coded factors, as follow:

Y1 (Q60) = 59.125 + 9.1113 × A [1] − 15.66 × A[2] + 18.042 × B − 5.594 × A[1]B + 6.9625 × A[2]B
Y2 (Q240) = 83.701 + 12.859 × A[1] − 21.58 × A[2] + 8.2778 × B − 2.753 × A[1]B + 6.6794 × A[2]B

Y3 (Fiber Size) = 647.96 − 115 × A[1] − 32.79 × [2] + 527.83 × B − 319.2 × A[1]B + 52.321 × A[2]B + 297.06 × B^2^
Y4 (Mucoadhesion Time) = 21.0542 − 0.02095 × A[1] + 0.02619 × A[2] − 11.7833 × B − 0.02857 × A[1]B + 0.03571 × A[2]B − 8.8375 × B^2^ (3)

where, A[1], A[2], and B are the coefficients of multi-level categorical and numerical factors.

#### 3.3.1. Solid State Characterization of RLX-Loaded Nanofibers

##### Scanning Electron Microscope (SEM) and Fiber Size

**Figure 1:**
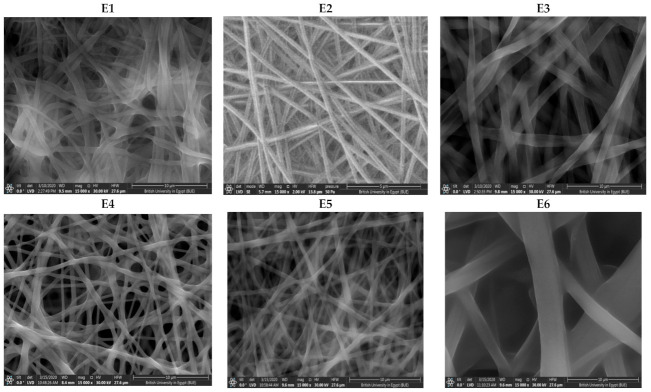

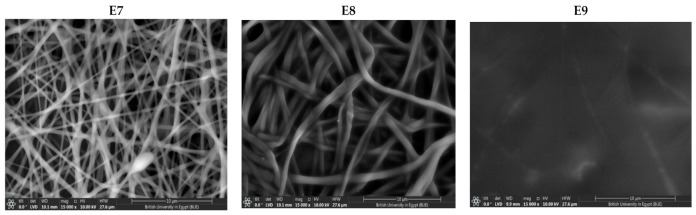
SEM of NFs formulae (**E1**–**E9**).

The SEM photographs and distribution of the average diameter are shown in Figure 1. SEM images of NFs films formulae E1-E8 revealed that the nanofibers were randomly aligned, interconnected and continuous. It can also be distinctly seen that smooth, bead-free, and relatively uniform fibrous diameter fibers were obtained. Since the drug was solubilized in a NE system containing a mixture of surfactant and co-surfactant, consequently it can be suggested that the NE played a role in decreasing the surface tension which enhances the electrospinnability of NFs. This comes in agreement with Wang et al. who observed that using non-ionic surfactant produced a smaller NFs that dramatically reduced surface tension than those obtained without surfactants [44].

Moreover, it was found that as the polymer to NE volume ratio increased, consequently the polymer concentration increased and resulted in enhanced electrospinnability of NFs and a uniform bead-free NFs is produced. Therefore, it could be observed that formulae with highest polymer ratio (E1, E4, and E7) produced smoother, bead-free, uniform diameter fibers. On the other hand, SEM images of E6 (least polymer:NE ratio) revealed less uniform, wider diameter NFs. Additionally, SEM images of E9 revealed that NFs were fused together which emerged as thin sheet in spite of a fibrous mat-like structure. Formulations E6 and E9 comprised least NE:polymer volume ratio (4:6) than other formulae.

Similar trends were observed by Jia et al. [45], who concluded that as the concentration of the polymer (CS/PVA) solution increased, beaded NFs transformed into uniform bead-free structure and also by Geng et al. [46], Who observed that the morphology of the nanofibers changed gradually from the highly beaded structure to uniform fiber structure with increasing the polymer concentration.

CS and HPMC pure solutions have poor electrospinning properties. Blending these biopolymers with PVA resulted in enhanced electrospinning properties which requires a higher PVA concentration in order to overcome the repulsive forces of the biopolymer molecules which prevent chains to be adequately entangled. On a study by Sedghi et al. [47] it was found that blending CS with PVA considerably enhanced its electrospinnability and subsequently produced NFs of uniform and smooth morphology [48,49].

Furthermore, Sedghi et al. found that as PVA concentration increased, the beads existed in NFs were completely resolved and simultaneously junction points or fused NFs disappeared. Similar results were observed by Aydogdu et al. and Lu et al. who found that non-homogeneous beaded NFs were obtained when pure HPMC was electrospun. Hence, polyethylene oxide was added to the HPMC solutions at different ratios. Due to the strong repulsive force between biopolymer molecules, sufficient chain entanglement cannot occur. Adding polyethylene oxide reduced the repelling force between molecules, promoting NFs formation [50,51].

As revealed in Table 1, the average diameters of the NFs ranged between 321.207 ± 26.14 nm (E7) to 1899.00 ± 2.9 nm (E9) associated with narrow size distribution and small standard deviations of NFs. Such results indicate that uniform NFs were obtained by electrospinning process [51]. The fiber size was significantly affected by NE:polymer ratio (*p* = 0.006). It was found that as the NE:polymer ratio increase, a significant increase in diameter of NFs was obtained.

Although the polymer concentration decreased as the NE:polymer volume ratio increased, the concentration of NE components (Miglyol^®^ 812 as an oil, Cremophor^®^ RH40 as surfactant and Transcutol^®^ HP as co-surfactant) increased which resulted in an increase in the solution viscosity. The diameter of fibers is directly proportional to the viscosity of the solutions. This was explained by Neo et al. [52] who proved that having high viscosity, prevented the instability of bending and reduced jet path. Due to the low stretching of the solutions, reducing the jet path resulted in high diameters. In a previous study, it was reported that increasing oil concentration during preparation of intranasal zaleplon NE from 10% to 20% resulted in a significantly larger globule size [27].

A direct correlation was observed between the fiber diameter and the type of polymer. PVA NFs have larger diameter than PVA/CS and PVA/HPMC-loaded NFs at the same NE:polymer volume ratio. This could be due to blending of polymers leads to smaller diameter than using one polymer. Similar observations have been made by Lin et al. [53] and Ignatova et al. [54] who investigated a series of blended NFs at different weight ratios and found a decrease in the average diameter of the NFs with increasing the blended content.

For PVA/CS NFs, the presence of amino groups in CS can be protonated in the acidic media. Therefore, the density of electrical charges on the surface of the jet increases in presence of CS than in PVA alone leading to higher charge density in the polymeric jet. Increasing the surface charge imposed greater elongation force on the jet under the electrical field, leading to decrease NFs size [49]. Similarly, Bahrami et al. found that finer fibers were produced with increasing CS content in the blend solution; on the other hand, fibers became more fragile [55].

##### Differential Scanning Calorimetry (DSC)

**Figure 2:**
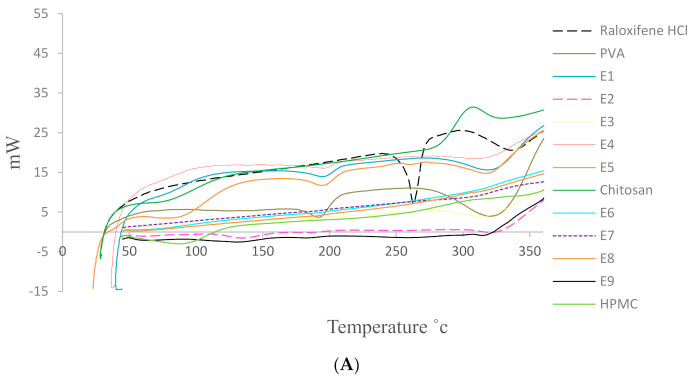

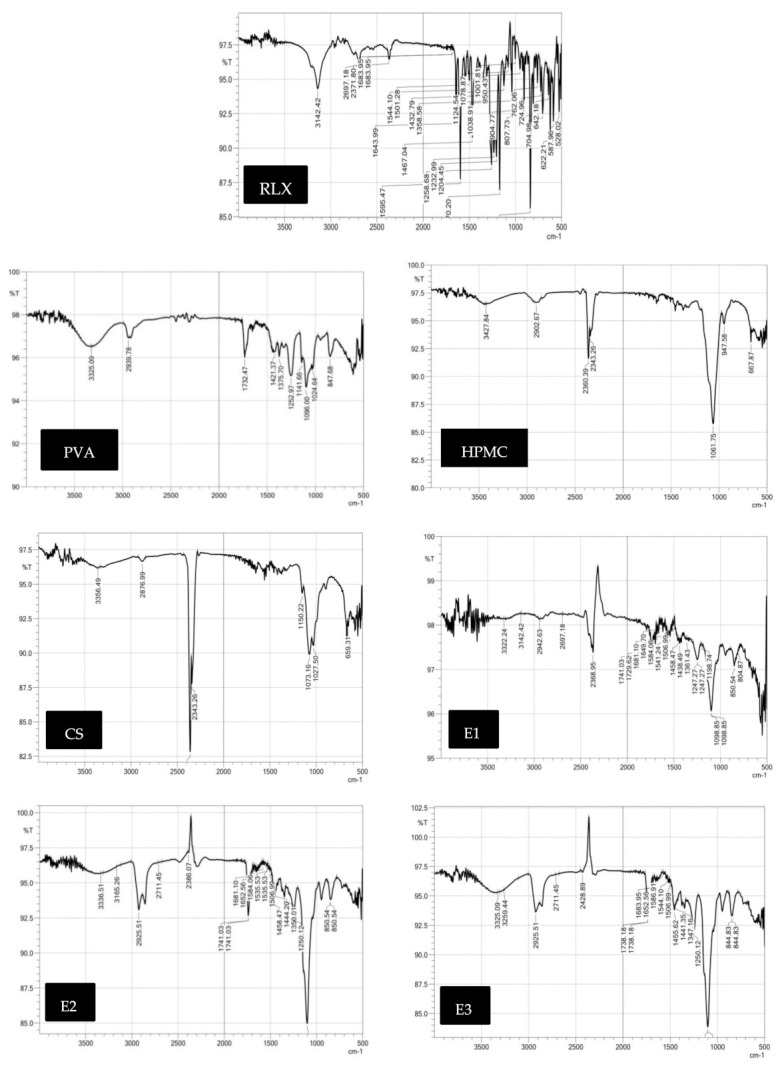

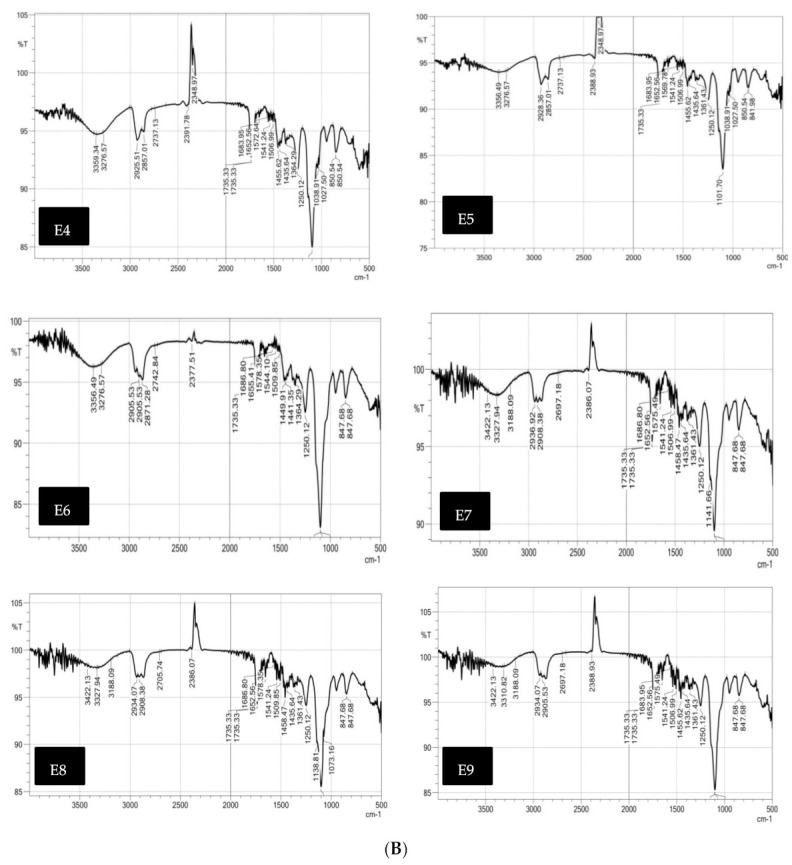
(**A**) DSC thermogram of RLX-NFs formulae (E1–E9) in comparison to that of the pure RLX, PVA, CS, and HPMC, (**B**) IR spectrum of pure (RLX, PVA, HPMC, CS, E1–E9).

The DSC thermograms of RLX, PVA, CS, HPMC, and RLX-loaded NF films are represented in Figure 2A. The DSC thermogram of RLX alone shows sharp endothermic peak at 262.71 °C which came in agreement with Patil et al. [56]. Such sharp peak is indicative of strong crystal lattice energy which can be considered as one of the factors responsible for lower RLX aqueous solubility [57].

Regarding the polymers, DSC thermogram of PVA showed two broad endothermic peaks at 194.24 °C and 325.19 °C corresponding to its melting points, which agrees with the finding of Salah et al. [58]. DSC thermogram of CS also presented broad endothermic peak at 79.22 °C that can be ascribed to the loss of water and a second exothermic peak at 305.69 °C that might be related to the decomposition of its amine unit according to Ferrero et al. [59]. Further, HPMC DSC thermograms showed a broad endothermic transition corresponding to its melting point. The endothermic transition of HPMC starts from 54.15 °C to 122.03 °C, with a broad peak value at 87.39 °C which is in agreement with Demappa et al. [60].

On the other hand, the sharp melting point peak of the drug completely disappeared in all RLX-NFs formulae, except for E5 where it was greatly decreased. Such disappearance of RLX characteristic peak could indicate the change of the crystalline drug into the amorphous state which might be molecularly dispersed throughout the developed NFs formulation. such changes in the drug state could contribute to the increase in RLX solubility and thus the enhanced dissolution profile [61].

##### Fourier-Transform Infra-Red Spectra (FT-IR)

The infrared spectrum of RLX, as shown in Figure 2B, exhibited the characteristic bands at 3142.42 cm^−1^ due to aromatic OH group, 2959.76 cm^−1^ due to CH aliphatic, 1680 cm^−1^ 1595.47 and 1432.79 cm^−1^ due to C=C, and 1258.68 cm^−1^ due to C–O stretching. 1170.20 cm^−1^ due to C–N and 850 cm^−1^ due to benzene ring para-substitution. For PVA, IR spectrum showed characteristic broad band at 3325.09 cm^−1^ corresponding to O–H alcoholic, band at 2939.78 cm^−1^ attributed to C–H SP3 and a band at 1089.38 corresponding to C–O–H. Additionally, HPMC IR spectrum showed characteristic broad band at 3427.84 cm^−1^ corresponding to O–H alcoholic, band at 2902.67 cm^−1^ attributed to C–H SP3 and a band at 1050 cm^−1^ corresponding to C–O. As for CS, IR spectrum showed characteristic biforked band at 3356.49 cm^−1^ corresponding to NH2 and a band at 2876.99 cm^−1^ attributed to O–H.

Alternatively, RLX-loaded NFs films IR spectrum showed decreased intensity in the characteristic bands of RLX. Most of RLX-peaks were found to be smoothened suggesting strong physical interaction with the polymers used [62] and the absence of formation of new peaks confirming the absence of any chemical interaction [63].

##### Powder X-ray Diffraction (PXRD) Studies

**Figure 3:**
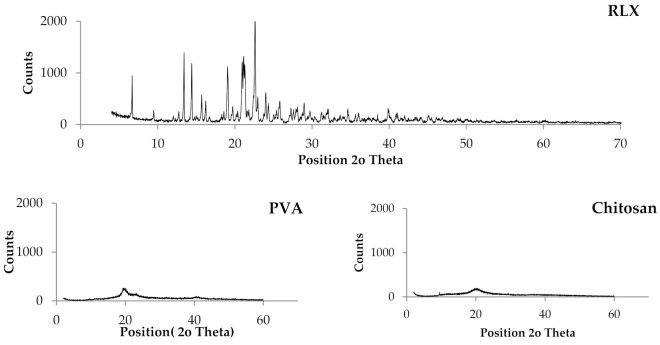

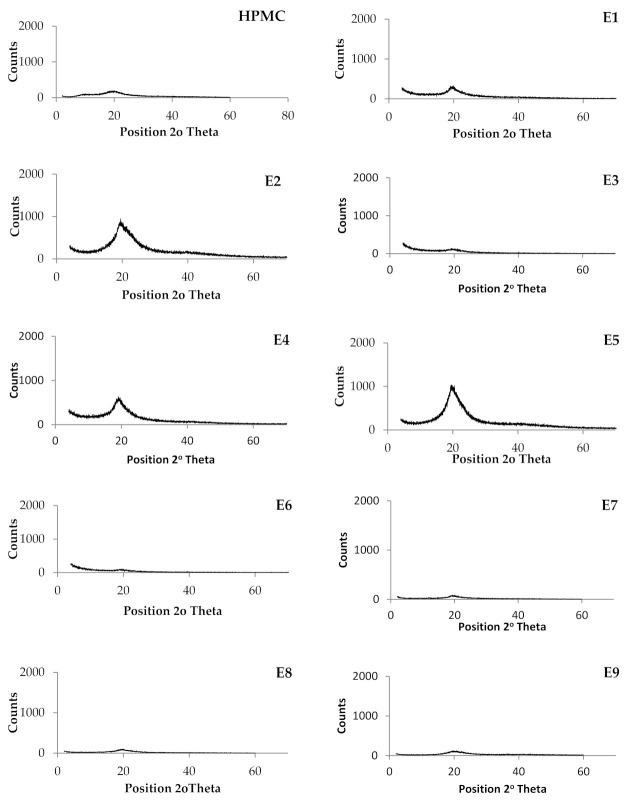
Powder X-ray diffraction of NFs formulae (E1–E9) in comparison to that of the pure (RLX, PVA, CS, HPMC).

The X-ray diffraction pattern for the pure drug, PVA, CS, HPMC, and RLX-loaded NF films are shown in Figure 3. The crystallinity of the phases influences the dissolution of the dosage form; an amorphous form dissolves at a faster rate relative to crystalline form because of its higher internal energy and greater molecular motion, which enhances the thermodynamic properties [64,65].

The PXRD pattern of the RLX exhibited sharp and intense peaks which is indicative of its strong crystalline nature with seven prominent peaks of high intensity at 2θ = 13.41°, 14.41°, 19.06°, 20.94°, 21.13°, 21.28°, and 22.62°. The other four less prominent peaks are at 2θ = 6.68°, 15.70°, 22.98°, and 24.01°.

Regarding the polymers used, all the polymer PXRD showed less sharp and intense peaks indicating lower crystallinity. The PXRD of PVA showed one prominent peak at 2θ = 19.65°. The PXRD of HPMC showed two prominent peaks at 2θ = 20° and 10.69°. The PXRD of CS showed one prominent peak at 2θ = 20.27°.

A comparison of PXRD of pure drug with that of RLX-loaded NFs films showed disappearance of all the drug peaks in all the formulae which could be attributed to the destruction of the drug crystal lattice because of progressive amorphization [66], which indicate that the drug might be molecularly dispersed inside the NF films which might lead to enhanced RLX solubility associated with augmented dissolution profile and bioavailability. It is worthy to note that such PXRD results are in great agreement with the DSC results.

#### 3.3.2. Determination of Drug Content and Homogeneity of RLX-Loaded Nanofibers

The mean values of RLX content in different RLX-loaded NFs films are presented in Table 1 and ranged from 92.38% to 105.33%. These results confirmed the absence of drug loss during the electrospinning procedures and uniform distribution of the drug through RLX-loaded NFs films.

#### 3.3.3. In Vitro Release Studies

**Figure 4:**
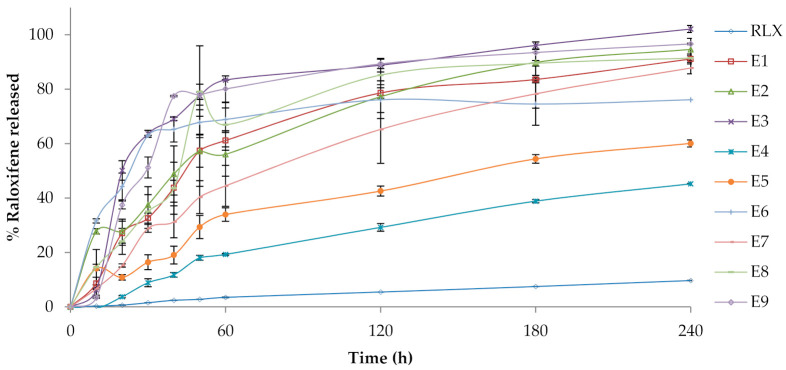
In vitro drug release profiles from RLX-loaded nanofibers (E1–E9) in comparison to RLX aqueous dispersion, in phosphate buffered saline (pH 6.8 of 0.1% *w*/*v* tween 80) at 37 ± 0.5 °C (mean ± S.D., *n* = 3).

The in vitro drug release profiles from RLX-loaded PVA, RLX-loaded PVA/HPMC and RLX-loaded PVA/CS core-sheath NFs in PBS pH 6.8 with 0.1% tween at 37 °C in comparison to RLX aqueous dispersion, are displayed in Figure 4. Practically, the burst drug release is required in order to rapidly achieve the minimum effective drug concentration, while the prolonged drug release is required to maintain the therapeutic drug response for an extended period [67].

A fast release of the drug was observed with an initial burst release of formulae E3, E6, E9, E2, E8, E1, and E7. This burst release could be attributed to the use of higher volume ratios of NE to polymer solution. As a result, core-sheath structure with an incomplete inward movement of the NE droplets during electrospinning process occurred. This allowed NE droplets to be located close to or loosely associated with the fiber surface or adsorbed on the fiber surface. Therefore, after 20 min, the initial burst release of the drug from NFs formulae with higher polymers ratio (E3(50.13%), E6(44.24%), and E9(37.43%)] was higher than NFs formulae with lower polymers ratio [E2(27.81%), E8(24.05), E1(27.07%), and E7(15.21%)). E4 and E5 showed (3.69%) and (10.83%) release of the drug respectively at time 20 min which could be attributed to the complete inward movement of NE inside the NFs. This comes in agreement with Yang, Y., Li et al. [68] who studied preparing proteins NFs using W/O emulsion-based electrospinning. They verified that the correlation between the volume ratio of aqueous to organic phase and burst release may be attributed to the completeness of core-sheath structure and unencapsulated proteins located close to, or loosely associated, with the fiber surface [68].

As well, O’Donnell et al. performed a study for evaluation of acetaminophen release from biodegradable PVA and nanocellulose films. It revealed that the fraction of burst release increased with increasing drug concentration in the initial formulations and this accounts for drug attached to the surface of the film and release, rather than being entrapped deeper in the polymer matrix [69].

Table 1 also presents the drug release profile discriminators (Q60 and Q240; response variables Y1 and Y2) of RLX-loaded NFs. Statistical analysis of the response variables confirmed that the type of polymer (A) and the NE:polymer ratio (B) had significant impacts on both Q60 and Q240.

Q60 at (*p* = 0.0097) and Q240 at (*p* = 0.0024) of RLX-loaded NFs significantly increased by increasing the NE:polymer ratio. As well, it was observed that for all the formulated NFs systems, increasing the volume ratio of NE to polymer solution resulted in a significant faster release from the NFs. This could be attributed to the fact that by increasing the ratio of NE to polymer solution, more drug will be in the NF films and also the polymer concentration will decrease, which results in a thinner shell phase and a faster release of the drug is observed. Yang et al. found that fibers prepared from a high volume ratio of aqueous to organic phase led to a reduction in the effective release lifetime, which may be due to the thinner shell phase of the matrix [68].

Additionally, the polymer type had significant effect on Q60 at (*p* = 0.0439) and Q240 at (*p* = 0.0005). As PVA, HPMC, and CS are hydrophilic biodegradable swellable polymers [70,71], when NFs are introduced in aqueous media, the possible mechanism of drug release is mostly through solvent penetration into the polymer matrix causing burst release, degradation (relaxation of the network) that creates more free volume for drug dissolution and drug diffusion to the surrounding medium. [72]. The free volume of a polymer matrix is dependent on its composition. Homogenous polymer solution exhibits increased free volume than heterogenous ones (i.e., polymeric mixture of PVA and HPMC or CS). This theory is based on the fact that there is an increased difference between ambient temperature and glass transition temperature in homogenous polymer solution. Consequently, drug diffusion and mobility are increased [73]. Additionally, as the viscosity of the polymer increases, the molecular mobility decreases and the diffusion of the drug through the NFs film decreases [72]. PVA has a viscosity of 55–65 cp, HPMC K100 LV has a viscosity of 100 cp, and CS has a viscosity of 800–2000 cp, therefore, PVA-loaded NFs show a faster release profile than PVA/HPMC and PVA/CS NFs due to higher drug mobility and diffusion through the NFs. PVA/HPMC-loaded NFs also showed faster release profiles than PVA/CS NFs. On top of that, the in vitro performance at PH 6.8 decreased CS solubility and the release of the drug decreased due to prolonged-diffusional release. That explains why the release of PVA-loaded NFs and PVA/HPMC-loaded NFs reported faster release than CS-loaded NFs at all NE-to-polymer ratios 1:9, 2:8 and 4:6.

Comparable results were introduced by O’Donnell et al. who found that control (PVA) films had the highest release, followed by other cellulose blended NF films due to the added resistance to the matrix [68].

#### 3.3.4. Bioadhesion Potential of RLX-Loaded NFs

Mucoadhesion strength of NFs films were measured by the mucoadhesion time that NFs films took to move from the top to the bottom of the agar/mucin gel plate. ANOVA results pointed out that NE:polymer ratio had significant effect on mucoadhesion time (Y4) with respective *p*-values of (*p* = 0.0001). An inverse correlation was established between NE:polymer ratio and mucoadhesion time, as the NE:polymer volume ratio decrease, (polymer concentration increase), a significant increase in the mucoadhesion time of NFs films was observed. Therefore, for the NFs formulae of high polymer concentrations, E1, E2, E4, E5, E7, and E8, the NFs mucoadhesion lasted till the end of the experiment (24 h). These results are on good agreement with Mishra et al. who developed a mucoadhesive buccal patches using 10% *w*/*v* PVA and 6% *w*/*v* HPMC and reported a mucoadhesion residence time more than 18 h [74].

On the other hand, for NFs E3, E6, and E9, the volume ratio of polymer:NE is 6:4, which has a lower polymer concentration than other NFs, consequently the mucoadhesion time of E3, E6, and E9 were 0.38, 0.5, and 0.42 h, respectively.

The concentration of the polymer is the mainstay factor affecting the mucoadhesion. It affects the development of a strong adhesive force with the mucus and it can be explained by the polymer chain length available for penetration into the mucus layer. When the concentration of the polymer is too low, the number of penetrating polymer chains per unit volume of the mucus is small, and the interaction between the polymer and mucus is unstable [75]. As the concentration of the polymer increase, it would result in a longer penetration chain length and better adhesion.

A direct correlation between the type of polymer and mucoadhesion time was observed. The mucoadhesion time of NFs E6 > E9 > E3. CS NFs (E6) has –OH and –NH2 groups leading to the formation of both hydrogen and covalent bonding. In addition, due to its positive charge, ionic interaction occurred with the negative charge of the sialic acid residues of mucus as well as epithelial surfaces [76]. HPMC NFs (E9) is proposed to form hydrogen bonding with the mucin present in the mucosal layer [77]. Nafee et al. found that mucoadhesive force of polyacrylic acid derivatives (PAA) showed the highest mucoadhesion, prolonged residence time. CS ensured promising mucoadhesive characteristics, whilst HPMC and exhibited weaker mucoadhesion [78].

### 3.4. Elucidation of Optimum RLX-NFs Films

Statistical optimization of the results was performed setting the optimization criteria for RLX-loaded NFs to minimize fiber size and maximize Q60, Q240, and mucoadhesion time. Formula E2 was chosen with the highest desirability value of (0.859). Hence, it was promoted for further investigations.

### 3.5. Characterization of the Optimum RLX-Loaded NFs

#### 3.5.1. HRTEM of the Selected RLX-Loaded NFs Film

The morphology of the NFs E2 was evaluated by high resolution TEM (HRTEM; JEM-2010, JEOL) with an accelerating voltage of 200 Kv. HRTEM was operated at 15 kV, and the fiber sample were prepared by making a suspension of the NFs in ethyl alcohol and directly depositing the as-spun ultrafine fibers suspension onto copper grids.

**Figure 5:**
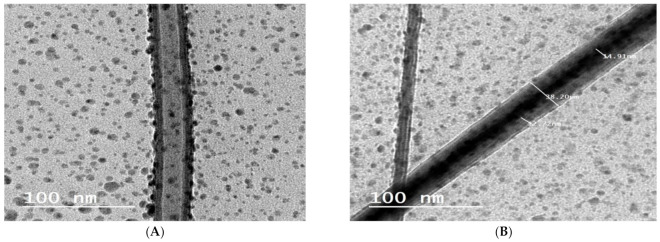
High resolution transmission electron micrographs (HRTEM) of the optimized formula (E2) showing; (**A**) core-sheath structure where a clear visual of the incorporated nanoemulsion is observed. Additionally, demonstrating the adsorption of some of the NE on the fiber surface. (**B**) NFs with clearly discriminated core and sheath with defined sizes.

HRTEM observation of the selected optimized NFs formula E2 was conducted to obtain an evidence that RLX-NE was indeed encapsulated within the shell material. It was observed that RLX-NE was wrapped into the center of the fibers (Figure 5). The sharp boundaries in the HRTEM images are indicative of the difference of electron transmission ability between the core and sheath materials [79]. As the NE emulsion exits the capillary of the electrospinner, stretching of the polymer and bending with the dispersed phase occurred, causing concentration of NE in the middle during incipient fiber elongation. During electrospinning, the emulsion droplets achieve their enrichment in the axial region and stretching into an oviform in the direction of the fiber path. The inward movement of emulsion droplets is due to the rapid elongation and evaporation of the solvents during the electrospinning. As a result of water evaporation, a rapid increase of the viscosity of the polymer solution was achieved. This viscosity gradient between the outer and the inner layer resulted in the inward movement of the NE droplets and, furthermore, the evaporation of water may cause their coalescence to some extent [80].

#### 3.5.2. Ex Vivo Drug Permeation Studies

**Figure 6:**
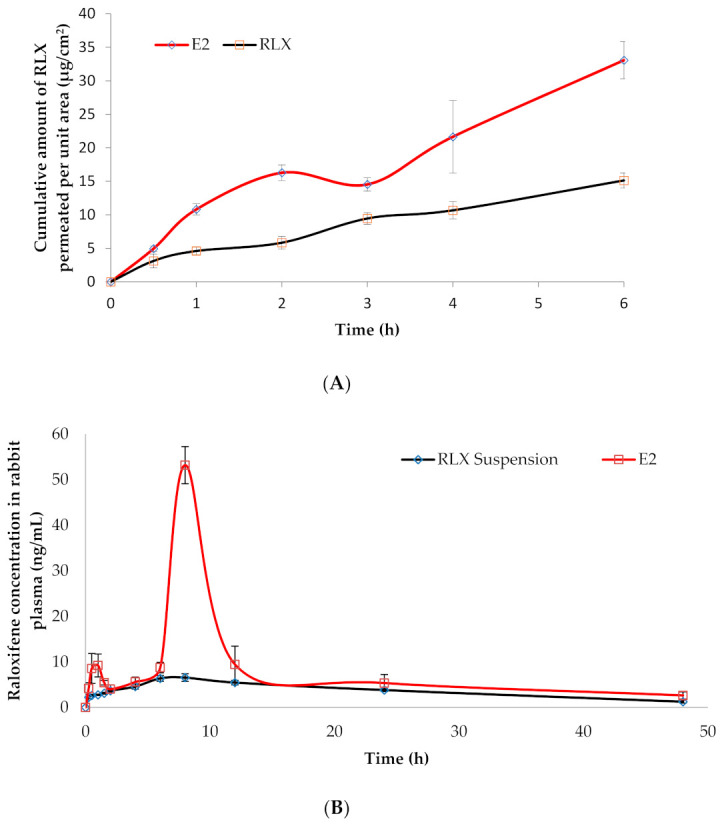
(**A**) Ex vivo drug permeation from RLX-loaded NFs formula (E2), in comparison to RLX aqueous dispersion, in phosphate buffer saline (pH 6.8 of 0.1% tween 80) at 37 ± 0.5 °C (mean ± SD; *n* = 3). (**B**) RLX plasma concentration-time profiles following the buccal application of RLX-loaded nanofibers formula (E2) and the oral administration of RLX aqueous dispersion in rabbits (mean ± S.D., *n* = 6).

The ex vivo drug permeation from the selected RLX-loaded NFs formula (E2) was studied and compared to its permeation from RLX aqueous dispersion and presented in Figure 6A. The permeation parameters studied were the cumulative amount permeated per unit area (Q6h), drug flux (Jss), and enhancement ratio (ER). It was clear that all the permeation parameters of RLX-NFs (E2) showed superiority over RLX aqueous dispersion. One way ANOVA revealed that NFs had significantly (*p* = 0.006) higher Q6h (33.06 ± 2.75 μg/cm^2^), significantly (*p* = 0.013) higher Jss (5.51 ± 0.45 μg/cm^2^/h) and significantly (*p* = 0.000) higher ER (2.12 ± 0.10) than RLX aqueous dispersion; Q6h (15.12 ± 1.12 μg/cm^2^), and Jss (2.52 ± 0.18 μg/cm^2^/h).

This could be explained by the extremely large surface area, unique surface topology, and porosity of the electrospun NFs, which significantly intensifies the intimate contact between the NF-based formula and mucosal surface, as well as the high drug concentration at the site of administration [17]. The influence of the NE system on the permeation of the loaded drug should be also kept into consideration; the NE’s nano-size and penetration-enhancing capability related to its component (surfactant; Cremophor RH 40^®^ and Transcutol HP^®^) greatly support and augment the drug permeation through the buccal mucosa [81,82].

### 3.6. In Vivo Estimation of RLX Pharmacokinetics in Rabbits

The RLX plasma concentration-time profiles following the buccal application of RLX-loaded NF films (E2; test treatment) and the oral administration of RLX aqueous suspension (reference treatment) in rabbits are displayed in Figure 6B and the observed RLX pharmacokinetic parameters were summarized in Table 2. Regular checking on the formulation in the buccal cavity of the rabbits was done to monitor the degradation progress. NFs kept their integrity up to 8 h with a decrease in the dimension as time elapsed.

The plasma concentration-time profiles of the test NFs (E2) formula showed a small peak after one hour which could be due to the burst release and rapid permeation by passive diffusion of the adsorbed, unloaded NE present on the surface of NFs [83]. Such results agree with the in vitro release results discussed earlier.

Statistical analysis of the pharmacokinetic parameters using multivariate ANOVA proved significant difference (*p* = 0.0001) between the C_max_ values of RLX following the oral administration of the reference treatment (7.04 ± 0.26 ng/mL) and the NFs formula E2 (53.18 ± 4.56 ng/mL).

The other hand, non-parametric Wilcoxon signed rank test revealed that both treatments had a non-significantly different median T_max_ of 8 h at (*p* = 1.00), which means that we retain the null hypothesis. These findings were in line with Shah et al. who reported T_max_ of RLX oral suspension to be 8 h [23]. This might be attributed to the gradual degradation of the multilayers of the NFs in order to release RLX-NE to be permeated through the buccal mucosa and the presence of the solid dispersed multilayered core-sheath NFs (Figures S1 and S2 in Supplementary Material). After layer-by-layer degradation of the NFs, release of NE occurred followed by passive diffusion of the NE.

Additionally, an insignificant difference was observed between the mean residence time (MRT_0–∞_) at (*p* = 0.580) and the elimination half-life (T_1/2_) at (*p* = 0.279) of RLX following the oral administration of the reference treatment (17.81 h ± 0.80 and 16.98 ± 3.31 h, respectively) and the buccal application of the test treatment; E2 (15.11 ± 1.70 h and 24.93 ± 11.51 h, respectively).

Taking into consideration the low oral bioavailability of RLX (2%) a significant improvement in the bioavailability of RLX was noted following the buccal application of the test treatment. The relative bioavailability of RLX was 2.29-fold higher (with respect to the mean AUC_0–48_ h values, (*p* = 0.001)) and 2.43-fold higher (with respect to the mean AUC_0–∞_ values, (*p* = 0.002)) (Table 2). The improved RLX bioavailability when formulated into NF buccal films could be attributed to several factors including, (i) enhancing the solubility of RLX via its formulation in the form of solid dispersed core-sheath electrospun NFs, (ii) the extremely large surface of NFs which promotes the intimate drug contact with the buccal mucosa promoting high drug concentration at the site of administration, (iii) the use Cremophor RH 40 and Transcutol HP in the formulation of the NE, which increases the permeation through the buccal mucosa, and finally (iv) the avoidance of the first pass metabolism due to buccal administration of the NFs films.

For the purpose of enhancing RLX bioavailability, Wang et al. [84] reported the formulation of RLX inclusion complex using sulfo-butyl-ether-β-cyclodextrin followed by conjugation to chitosan to form RLX-loaded cyclodextrin/chitosan nanoparticles (RLX-ccNPs). The enhancement of oral bioavailability of RLX-ccNPs relative to RLX suspensions was about 3.60-fold. Saini et al. [26] developed intranasal RLX-loaded chitosan nanoparticles, which enhance RLX bioavailability by ~1.7-fold. Murthy et al. [22] developed soy lecithin-chitosan complex nanoparticles (LCNPs) for oral delivery of RLX. After loading RLX into LCNPs, its bioavailability increased significantly (*p* < 0.05) by over ~4.2-fold compared to the free drug suspension. The observed 2.43-fold higher AUC_0–∞_ values of the developed NFs demonstrated how this technique established itself among other nanocarrier systems. Additionally, NFs films present the advantages of solid dosage forms over nanocarrier dispersion systems: Stability, easy handling, increased patient compliance, and accurate dosing.

## 4. Conclusions

Solid dispersed multilayered core-sheath RLX-loaded NFs films were successfully developed utilizing the nanoemulsion electrospinning technique without the use of organic solvents, which paves the road to ‘‘green’’ drug delivery systems. This would vastly extend their potential in therapy. According to the D-optimal response surface methodology, the investigated variables included the NE:polymer ratio and polymer type. Considering the Q60, Q240, fiber size, and mucoadhesion time, the highest desirability values were achieved with E2, comprising RLX-NE and NE:polymer (PVA) at a ratio of 2:8. The superiority of E2 over RLX aqueous dispersion was revealed with respect to the ex vivo drug permeation parameters, Q6h, Jss, and ER and improved bioavailability of RLX by ≈ 2.29-fold. RLX-loaded NFs (E2) would be expected to rapidly initiate and maintain the therapeutic response of RLX for a sufficient period, thus enhancing RLX bioavailability. Further clinical studies can be conducted to confirm these findings.

## Figures and Tables

**Table 1:** The composition and in vitro characterization results (mean ± S.D., *n* = 3) of the developed raloxifene nanofibers.

System	Composition (mL)				In Vitro Characterization Data of RLX-NFs				
	RLX-NE (5 mg/mL)	PVA (10% w)	HPMC (1% w)	Chitosan (1.5% *w*/*v*)	Drug Content (%)	Mucoadhesion Time (h)	Fiber Size (nm)	Q60 * (%)	Q240 * (%)
E1	1	9	-	-	99.90 ± 5.52	24 ± 0.00	555.82 ± 29.88	61.14 ± 7.5	91.10 ± 3.63
E2	2	8	-	-	96.56 ± 0.62	24 ± 0.00	594.67 ± 26.63	56.04 ± 16.02	94.61 ± 8.16
E3	4	6	-	-	102.45 ± 4.98	0.38 ± 0.01	1005.93 ± 3.52	83.36 ± 0.14	102.11 ± 2.61
E4	1	7.2	-	1.8	105.33 ± 7.10	24 ± 0.00	374.54 ± 20.74	19.26 ± 0.65	45.20 ± 0.31
E5	2	6.4	-	1.6	98.20 ± 14.62	24 ± 0.00	391.10 ± 15.42	33.91 ± 4.93	60.06 ± 2.60
E6	4	4.8	-	1.2	97.31 ± 0.80	0.5 ± 0.07	1513.61 ± 46.23	68.87 ± 9.29	76.09 ± 15.59
E7	1	7.2	1.8	-	102.33 ± 6.59	24 ± 0.00	321.20 ± 26.14	44.47 ± 7.53	87.76 ± 2.10
E8	2	6.4	1.6	-	93.38 ± 7.03	24 ± 0.00	529.27 ± 79.49	66.92 ± 16.26	91.43 ± 1.77
E9	4	4.8	1.2	-	92.38 ± 2.14	0.42 ± 0.05	1899 ± 2.19	80.08 ± 9.59	96.62 ± 0.57

* Q60 and Q240 represent the % drug released after 60 min and 240 min. RLX-NE; raloxifene nanoemulsion, PVA; polyvinyl alcohol and HPMC; hydroxypropyl methylcellulose.

**Table 2:** The pharmacokinetic parameters and relative bioavailability of RLX following buccal application of RLX-loaded NF (E2) films and oral administration of RLX aqueous dispersion in rabbits (mean ± S.D., *n* = 6)

Treatments	Oral RLX Aqueous Dispersion	RLX-Loaded NF (E2) Buccal Film
C_max_ (ng/mL)	7.04 ± 0.26	53.18 ± 4.56
T_max_ (h) *	8	8
MRT_0–∞_ (h)	17.81 ± 0.80	15.11 ± 1.70
t_1/2_ (h)	16.98 ± 3.31	24.93 ± 11.51
AUC_0–48_ (ng·h/mL)	177.92 ± 11.51	408.74 ± 59.21
AUC_0–∞_ (ng·h/mL)	209.61 ± 22.20	509.84 ± 71.72
% relative bioavailability based on AUC _(0–48)_		229.73
% relative bioavailability based on AUC _(0–∞)_		243.22

* Median (range).

## Data Availability

All data generated or analyzed during this study are included in this published article (and its Supplementary material file).

## Supplementary Materials

The following are available online at https://www.mdpi.com/article/10.3390/pharmaceutics13040474/s1, Figure S1: Nanofiber film (E2) showing multilayered layout, Figure S2: A photo of nanofibers film E2, Table S1: Non-parametric test summary, Table S2: Related-samples Wilcoxon signed rank test summary, Table S3: Between-subjects factors, Table S4: Multivariate tests, Table S5: Tests of between-subjects effects.
