# Supplementary material for: Green Nanotechnology in the Formulation of a Novel Solid Dispersed Multilayered Core-Sheath Raloxifene-Loaded Nanofibrous Buccal Film; In Vitro and In Vivo Characterization
Source: Pharmaceutics. 2021 Apr 1;13(4):474. doi: 10.3390/pharmaceutics13040474 (PMC8066100; doi:10.3390/pharmaceutics13040474)
Supplement: Supplementary file 1 [file pharmaceutics-13-00474-s001.pdf]

pharmaceutics

Supplementary Materials: Green Nanotechnology in the Formulation of a Novel Solid Dispersed Multilayered Core-Sheath Raloxifene-Loaded Nanofibrous Buccal Film; In Vitro and In Vivo Characterization

Sara Nageeb El-Helaly, Eman Abd-Elrasheed, Samar A. Salim, Rania H. Fahmy, Salwa Salah and Manal M. EL-Ashmoony

Table S1. Non-parametric Test Summary.

	Null Hypothesis	Test	Sig.[a,b]	Decision
1	The median of differences between Tmax for Raloxifene Suspension and Tmax for Nanofibers equals 0.	Related-Samples Wilcoxon Signed Rank Test	1.000	Retain the null hypothesis.

a. The significance level is 0.050, b. Asymptotic significance is displayed.

Table S2. Related-Samples Wilcoxon Signed Rank Test Summary.

Total N	5
Test Statistic	0.000
Standard Error	0.000
Standardized Test Statistic	0.000
Asymptotic Sig.(2-sided test)	1.000

Table S3. Between-Subjects Factors.

		Value Label	N
Rabbits	1		2
	2		2
	4		2
	5		2
	6		2
The type of formulation prepared	1	Raloxifene Suspension	5
	2	Nanofibers	5
Period	1	Period 1	5
	2	Period 2	5

Table S4. Multivariate Tests[a.]

Effect		Value	F	Hypothesis df	Error df	Sig.
Intercept	Pillai's Trace	1.000	20080.776 [b]	3.000	1.000	0.005
	Wilks' Lambda	0.000	20080.776 [b]	3.000	1.000	0.005
	Hotelling's Trace	60242.327	20080.776 [b]	3.000	1.000	0.005
	Roy's Largest Root	60242.327	20080.776 [b]	3.000	1.000	0.005
Rabbits	Pillai's Trace	2.032	1.574	12.000	9.000	0.252
	Wilks' Lambda	0.001	2.743	12.000	2.937	0.224
	Hotelling's Trace	.	.	12.000	.	.
	Roy's Largest Root	169.159	126.869 [c]	4.000	3.000	0.001

Formulation	Pillai's Trace	0.940	5.215 [b]	3.000	1.000	0.309
	Wilks' Lambda	0.060	5.215 [b]	3.000	1.000	0.309
	Hotelling's Trace	15.645	5.215 [b]	3.000	1.000	0.309
	Roy's Largest Root	15.645	5.215 [b]	3.000	1.000	0.309
Period	Pillai's Trace	0.992	40.100 [b]	3.000	1.000	0.115
	Wilks' Lambda	0.008	40.100 [b]	3.000	1.000	0.115
	Hotelling's Trace	120.299	40.100 [b]	3.000	1.000	0.115
	Roy's Largest Root	120.299	40.100 [b]	3.000	1.000	0.115

a. Design: Intercept + Rabbits + Formulation + Period, b. Exact statistic, c. The statistic is an upper bound on F that yields a lower bound on the significance level.

Table S5. Tests of Between-Subjects Effects.

Source	Dependent Variable	Type II Sum of Squares	df	Mean Square	F	Sig.
Corrected Model	Elimination rate constant	0.001 [a]	6	0.000	0.584	0.737
	Elimination half life	431.056 [b]	6	71.843	0.716	0.668
	Mean residence Time	328.828 [c]	6	54.805	0.497	0.786
	ln transformed Cmax	10.217 [d]	6	1.703	357.655	0.000
	ln transformed AUC0t	1.776 [e]	6	0.296	31.305	0.008
	ln transformed AUC infinity	2.031 [f]	6	0.338	22.576	0.014
Intercept	Elimination rate constant	0.014	1	0.014	53.227	0.005
	Elimination half life	4393.216	1	4393.216	43.786	0.007
	Mean residence Time	7774.550	1	7774.550	70.482	0.004
	ln transformed Cmax	87.678	1	87.678	18415.706	0.000
	ln transformed AUC0t	312.720	1	312.720	33081.336	0.000
	ln transformed AUC infinity	334.525	1	334.525	22315.857	0.000
Rabbits	Elimination rate constant	0.001	4	0.000	0.708	0.638
	Elimination half life	253.840	4	63.460	0.632	0.674
	Mean residence Time	277.094	4	69.274	0.628	0.676
	ln transformed Cmax	0.021	4	0.005	1.124	0.481
	ln transformed AUC0t	0.063	4	0.016	1.664	0.352
	ln transformed AUC infinity	0.067	4	0.017	1.110	0.485
Formulation	Elimination rate constant	0.000	1	0.000	0.503	0.529
	Elimination half life	174.013	1	174.013	1.734	0.279
	Mean residence Time	42.320	1	42.320	0.384	0.580
	ln transformed Cmax	9.774	1	9.774	2053.013	0.000
	ln transformed AUC0t	1.582	1	1.582	167.357	0.001
	ln transformed AUC infinity	1.851	1	1.851	123.487	0.002
Period	Elimination rate constant	1.848×10^{-5}	1	1.848×10^{-5}	0.068	0.811
	Elimination half life	19.289	1	19.289	0.192	0.691
	Mean residence Time	18.553	1	18.553	0.168	0.709
	ln transformed Cmax	0.000	1	0.000	0.023	0.889
	ln transformed AUC0t	0.011	1	0.011	1.113	0.369
	ln transformed AUC infinity	0.003	1	0.003	0.216	0.673
Error	Elimination rate constant	0.001	3	0.000		
	Elimination half life	300.999	3	100.333		
	Mean residence Time	330.918	3	110.306		
	ln transformed Cmax	0.014	3	0.005		
	ln transformed AUC0t	0.028	3	0.009		
	ln transformed AUC infinity	0.045	3	0.015		
Total	Elimination rate constant	0.016	10			
	Elimination half life	5125.271	10			

	Mean residence Time	8434.297	10		
	ln transformed Cmax	97.909	10		
	ln transformed AUC0t	314.524	10		
	ln transformed AUC infinity	336.600	10		
Corrected Total	Elimination rate constant	0.002	9		
	Elimination half life	732.055	9		
	Mean residence Time	659.747	9		
	ln transformed Cmax	10.231	9		
	ln transformed AUC0t	1.804	9		
	ln transformed AUC infinity	2.075	9		

a. R Squared = 0.539 (Adjusted R Squared = −0.384), b. R Squared = 0.589 (Adjusted R Squared = −0.234), c. R Squared = 0.498 (Adjusted R Squared = −0.505), d. R Squared = 0.999 (Adjusted R Squared = 0.996), e. R Squared = 0.984 (Adjusted R Squared = 0.953), f. R Squared = 0.978 (Adjusted R Squared = 0.935).

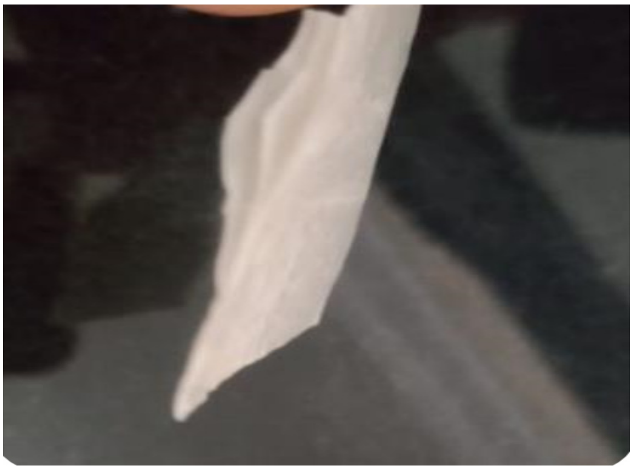

Figure S1. Nanofibers film (E2) showing Multilayered layout.

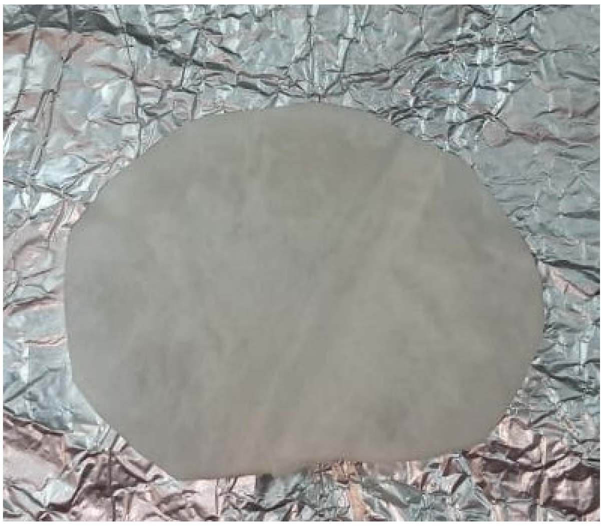

Figure S2. A photo of Nanofibers film E2.

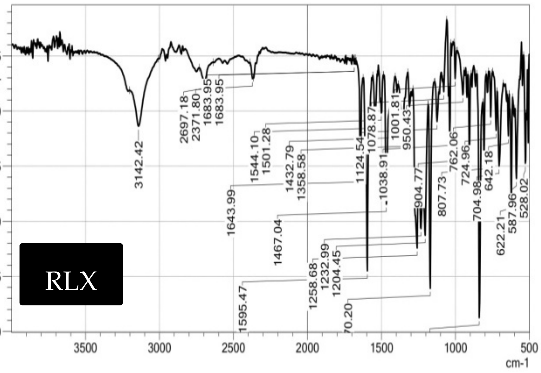
RLX
%T
cm-1

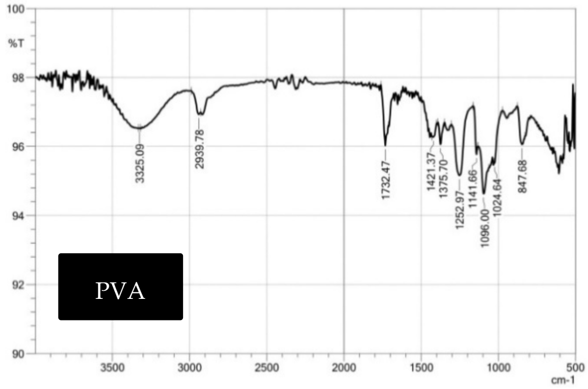
PVA
%T
cm-1

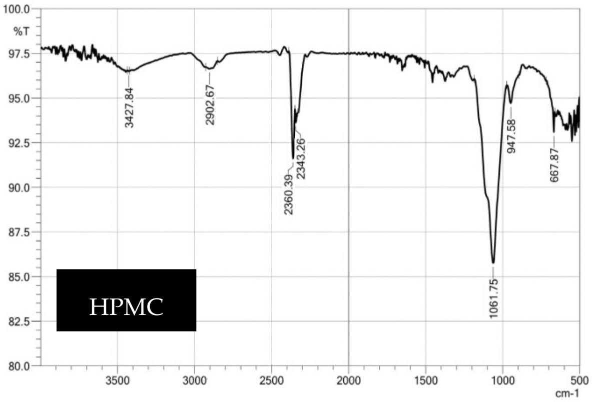
HPMC
%T
cm-1

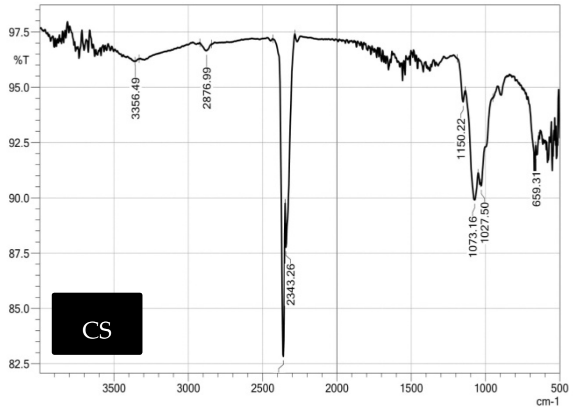
CS
%T
cm-1

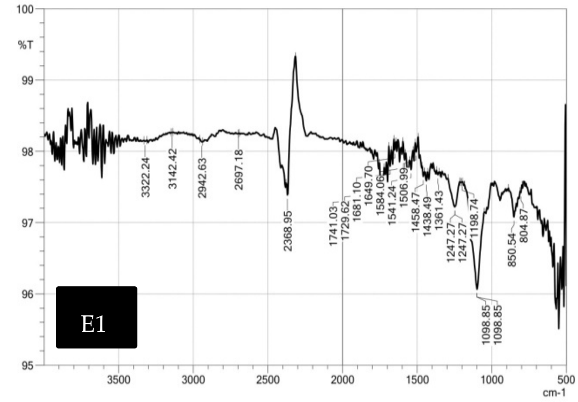
E1
%T
cm-1

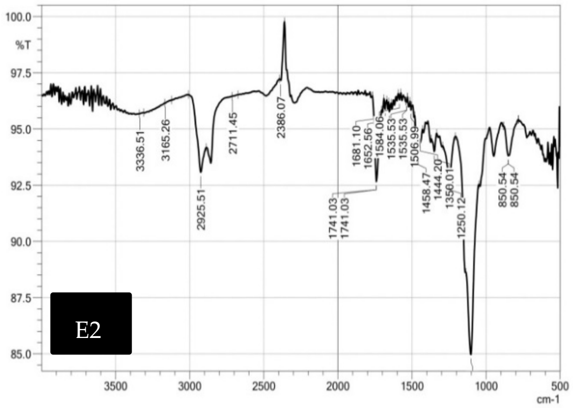
E2
%T
cm-1

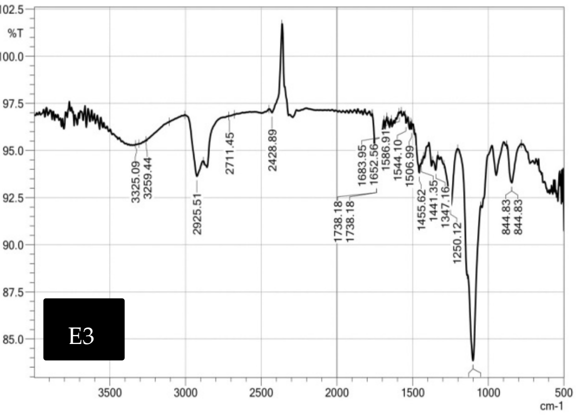
E3
%T
cm-1

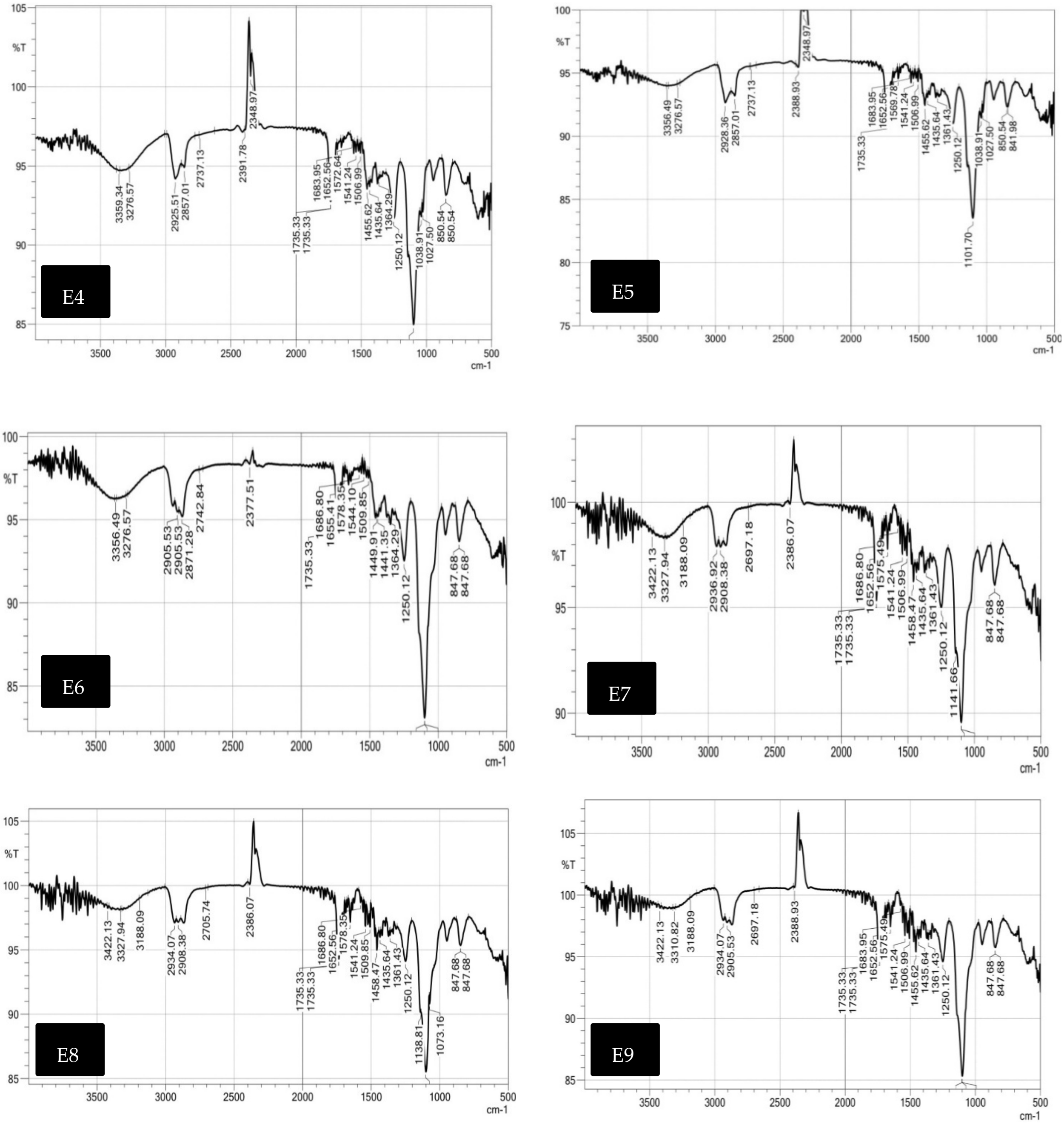

Figure S3. IR spectrum of pure RLX, PVA, HPMC, CS, E1, E2, E3, E4, E5, E6, E7, E8, E9.
